# Machine learning and experiments identifies SPINK1 as a candidate diagnostic and prognostic biomarker for hepatocellular carcinoma

**DOI:** 10.1007/s12672-023-00849-2

**Published:** 2023-12-14

**Authors:** Shiming Yi, Chunlei Zhang, Ming Li, Tianyi Qu, Jiafeng Wang

**Affiliations:** 1 Department of Hepatobiliary Surgery, Yantai Affiliated Hospital of Binzhou Medical University, Yantai, China; 2 Department of Colorectal and Anus Surgery, Yantai Affiliated Hospital of Binzhou Medical University, Yantai, China; 3 Department of Gastroenterology, Yantai Affiliated Hospital of Binzhou Medical University, Yantai, China; 4 Emergency Department, Yantai Affiliated Hospital of Binzhou Medical University, Yantai, China; 5 Department of Hepatobiliary Surgery, the Affiliated Taian City Central Hospital of Qingdao University, Taian, China

**Keywords:** Hepatocellular carcinoma, Immune infiltration, Biomarker, SPINK1, Diagnosis, Machine learning, Wnt/β-catenin pathway

## Abstract

Machine learning techniques have been widely used in predicting disease prognosis, including cancer prognosis. One of the major challenges in cancer prognosis is to accurately classify cancer types and stages to optimize early screening and detection, and machine learning techniques have proven to be very useful in this regard. In this study, we aimed at identifying critical genes for diagnosis and outcomes of hepatocellular carcinoma (HCC) patients using machine learning. The HCC expression dataset was downloaded from GSE65372 datasets and TCGA datasets. Differentially expressed genes (DEGs) were identified between 39 HCC and 15 normal samples. For the purpose of locating potential biomarkers, the LASSO and the SVM-RFE assays were performed. The ssGSEA method was used to analyze the TCGA to determine whether there was an association between SPINK1 and tumor immune infiltrates. RT-PCR was applied to examine the expression of SPINK1 in HCC specimens and cells. A series of functional assays were applied to examine the function of SPINK1 knockdown on the proliferation of HCC cells. In this study, 103 DEGs were obtained. Based on LASSO and SVM-RFE analysis, we identified nine critical diagnostic genes, including C10orf113, SPINK1, CNTLN, NRG3, HIST1H2AI, GPRIN3, SCTR, C2orf40 and PITX1. Importantly, we confirmed SPINK1 as a prognostic gene in HCC. Multivariate analysis confirmed that SPINK1 was an independent prognostic factor for overall survivals of HCC patients. We also found that SPINK1 level was positively associated with Macrophages, B cells, TFH, T cells, Th2 cells, iDC, NK CD56bright cells, Th1 cells, aDC, while negatively associated with Tcm and Eosinophils. Finally, we demonstrated that SPINK1 expression was distinctly increased in HCC specimens and cells. Functionally, silence of SPINK1 distinctly suppressed the proliferation of HCC cells via regulating Wnt/β-catenin pathway. The evidence provided suggested that SPINK1 may possess oncogenic properties by inducing dysregulated immune infiltration in HCC. Additionally, SPINK1 was identified as a novel biomarker and therapeutic target for HCC.

## Introduction

Hepatocellular carcinoma (HCC) is acknowledged as the third common malignant tumor worldwide, and the primary contributors of HCC are conditions such as alcoholic liver disease, non-alcoholic fatty liver disease and viral infections with hepatitis B and C [1, 2]. Patients who were identified with advanced HCC did not obtain therapies that were beneficial, despite the fact that breakthroughs in surgery and chemotherapy had significantly improved the prognosis of patients with HCC [3, 4]. The most common reason for a poor prognosis and unsuccessful treatment options for HCC patients is the development of metastatic disease [5, 6]. Extensive research over the past few decades into the molecular processes underlying the pathogenesis of HCC has uncovered the roles that gene mutations, epigenetic changes, and dysregulation of coding and non-coding genes play in controlling HCC development [7, 8]. However, the sensitive biomarkers for diagnosis and prognosis of HCC were rarely reported. The prognostic prediction of HCC is difficult because of the numerous complicated etiologic variables, as well as the high-level heterogeneity of the disease. In addition, taking into account the restricted therapeutic options for HCC, there is an extra need for the creation of innovative prognostic biomarkers.

Machine learning has become a powerful tool in the field of cancer research, particularly in identifying diagnostic genes [9]. The process of screening diagnostic genes can be a difficult and time-consuming effort; however, machine learning algorithms have the potential to assess vast volumes of data in a relatively short amount of time [10]. This might make the screening of diagnostic genes a more feasible option. These algorithms are able to recognize patterns and correlations within genomic data that are difficult for people to recognize on their own [11, 12]. Researchers are able to filter massive collections of genetic data and uncover prospective diagnostic genes that may be connected with certain forms of cancer by utilizing machine learning. This strategy may result in the diagnosis of cancer at an earlier stage, which will ultimately lead to improved patient outcomes [13, 14]. Traditional screening procedures, which are prone to subjectivity and human error, can be improved with the use of machine learning, which can also help overcome some of the limits of such approaches. Machine learning is able to uncover patterns and trends within genetic data that are not immediately obvious to the naked eye by making use of powerful algorithms. The new methods have the potential to assist in the identification of critical biomarkers that are linked with various cancer types and has the potential to give vital insights into the underlying processes of the illness [15, 16]. Machine learning may also be used to construct predictive models, which can assist identify people who are at a greater risk of acquiring cancer. These models can be used to better target cancer prevention and treatment efforts. In general, the application of machine learning in the field of cancer research has the potential to transform our understanding of the illness and to improve the prognosis of patients by enabling earlier identification and more individualized treatment choices.

The immune microenvironment plays a crucial role in tumor development and progression. Tumors arise from the uncontrolled proliferation of abnormal cells, and the immune system is responsible for recognizing and eliminating these abnormal cells [17, 18]. However, cancer cells can evade the immune system by developing mechanisms to suppress the immune response. Immune cells, cancer cells, and stromal cells are all examples of what are included in the immunological microenvironment [19, 20]. The term refers to the cells and substances that make up the immediate environment of the tumor. These cells and chemicals work together to form a complex network that may either encourage or suppress the growth of tumors depending on how they interact with one other. Other cells, such as myeloid-derived suppressor cells and regulatory T cells, can inhibit the immune response and encourage tumor growth. Cancer cells can be recognized and attacked by immune cells such as natural killer cells, B cells and T cells [21, 22]. In order to create successful cancer treatments that can tap into the capacity of the immune system to combat cancer, it is essential to have a solid understanding of the intricate interactions that take place inside the immune microenvironment. In addition to its roles in tumor development and progression, the immune microenvironment also plays a crucial role in predicting patient outcomes and response to therapy. Growing researches have shown that tumors with higher expressions of immune cell infiltration, particularly cytotoxic T cells, are associated with better patient outcomes and response to immunotherapy [23, 24]. On the other hand, tumors with low immune cell infiltration are often resistant to immunotherapy and associated with poor patient outcomes. Therefore, assessing the immune microenvironment of tumors is becoming increasingly important for personalized cancer treatment [25, 26]. Various techniques, such as immune profiling and immune monitoring, are used to analyze the immune microenvironment of tumors and identify potential targets for therapy.

Using the GEO database and the TCGA datasets, we obtained two microarray datasets relating to HCC and downloaded them. An investigation into the genes that were differentially expressed between the HCC and the controls was carried out. For the purpose of filtering and identifying HCC diagnostic biomarkers, machine-learning techniques were utilized. In addition, it was the first time that ssGSEA was used to determine the proportions of immune cells in samples of HCC and normal specimens based on the gene expression profile of these samples. Furthermore, we explored potential function of the critical genes in HCC.

## Materials and methods

### Patients

A total of 15 HCC specimens and matched non-tumor specimens were randomly obtained from samples that were removed from patients who were treated in the Affiliated Taian City Centeral Hospital of Qingdao University between 2021 and 2022. The fresh tissue samples were flash-frozen in liquid nitrogen as soon as they were collected. Before undergoing surgery, not one of these individuals had been treated with radiation or chemotherapy. The study was approved by the Ethics Committee of the Affiliated Taian City Centeral Hospital of Qingdao University and was conducted in compliance with the Helsinki Declaration. Informed consent was obtained from every patient.

### Cell culture and transfection

HCC Cells (MHCC97H, HepG2, HCCLM3 and SMMC-7721), and LO2 cells (as control cells) were bought from Bena Culture Collection (Kunshan, Jiangsu, China). They were grown in RPMI-1640 medium (with 10% FBS). The conditions for the cell culture were as follows: 37 degrees Celsius and 5% CO_2_. The shRNAs (shRNA-NC, shRNA-SPINK1-1 and shRNA-SPINK1-2) were purchased from JiMa Biological corporation. In line with the methods that were included in the Lipofectamine 2000 reagent kits, the cell transfection was carried out utilizing those kits.

### RNA extraction and qRT-PCR analysis

Using a Total RNA extraction kit, RNA was taken from the cells of both cell lines in order to complete the extraction process. Sangon Biotech Co., Ltd. was responsible for the conception and synthesis of the primer sequences for both SPINK1 and GAPDH. Following the instructions provided by the manufacturer of the Reverse Transcriptase Kit (Beyotime Institute of Biotechnology), total RNA was converted into cDNA by the process of reverse transcription. Then, qRT-PCR was performed using SYBR Premix Ex Taq II (Takara Biotechnology, China). The primer sequences were as follows: SPINK1-forward, 5′- TCTATCTGGTAACACTGGAGCTG-3′; reverse, 5′- ACACGCATTCATTGGGATAAGT-3′; GAPDH-forward, 5′- CTGGGCTACACTGAGCACC-3′; reverse, 5′-AAGTGGTCGTTGAGGGCAATG-3′. The PCR was carried out in a reaction mixture of 10 µL, and the protocol called for an initial denaturation step at 95 °C for 30 s, followed by amplification with 40 cycles at 95 °C for 5 s and 60 °C for 30 s; this was followed by a melt curve step at 65 °C to 95 °C, with temperature increments of 0.5 °C for 5 s. GAPDH was utilized throughout this study as the endogenous control. The 2-Ct technique was utilized in the analysis of the data pertaining to gene expression.

### Cell counting kit-8 (CCK-8) assay

Using the CCK-8 kit, we were able to evaluate the cell viability (Dojindo Molecular Technologies, Inc., Shanghai, China). Following the completion of the transfections, 96-well plates were seeded with 100 µL of cells at a density of 5 × 10^3^ cells per well. At the 0 h, 24 h, 48 h, and 72 h marks, 10 L of the CCK-8 solution was added to each well.

### EdU incorporation assay

The EdU incorporation test kit (RiboBio, China) was used to conduct the EdU proliferation assays. In 96-well plates, transfected cells at a density of 2 × 10^4^ cells per well were planted. After a total of 24 h, 100 ul of media containing 50 mM EdU was added to each well, and the plates were put into an incubator for a period of two hours, during which time the temperature was maintained at 37 degrees Celsius. After this step, the cells were stained with Hoechst and an Apollo reaction cocktail and then fixed with 4% paraformaldehyde.

### Colony-formation assay

Following the completion of the standard incubation period, the transfected cells were trypsinized, centrifuged, counted, and then replanted at a density of 500 cells per 6 cm plate. Following a period of 12 days, the cell colonies were preserved with methanol at a concentration of 37%, stained with crystal violet, and counted using a microscope. Each cell colony included at least 50 cells.

### Western blot analysis

The cells in each group were subjected to a variety of treatments for a period of 48 h, after which they were collected and centrifuged at room temperature for 24 h in order to remove the supernatant. Following the lysis of the cells using cell lysates, the supernatant was collected for examination. The BCA solution was used to determine the amount of protein that was present in the supernatant. After 12% SDS-PAGE gel electrophoresis, the proteins in the gel were transferred to PVDF membranes at a rate of 60 g per lane. The membranes were then blocked with 5% skim milk powder for two hours at 4 degrees Celsius. And then incubated with primary antibodies against SPINK1, β-catenin and Actin at room temperature for 3 h, and then incubated with horseradish peroxidase‐conjugated goat anti‐rabbit secondary antibody at room temperature for 1 h. Abcam served as the source for all of the antibodies. Then, the membranes were washed with TBST five times for ten minutes at a time, and then protein bands were seen with ECL luminous fluid. Within this study, a decision was made to employ a membrane cropping strategy driven by cost-effectiveness considerations. This choice was rooted in the constraints posed by limited experimental materials and resources, coupled with the necessity to ensure the feasibility of the experiments. Despite the adoption of a streamlined membrane cropping approach in image processing, we have maintained result clarity and interpretability by showcasing the membrane edges in each blot image.

### Mouse tumor xenograft model

The Shanghai Slark Experimental Animal Company provided the nude female BALB/c mice, which ranged in age from 5–6 weeks to 15–20 g and were maintained in settings that were pathogen-free. Randomization was used to divide the animals into two groups, each containing six mice. After placing HepG2 cells in phosphate-buffered saline at a concentration of 2 × 10^7^/mL, the mice all had 100 L of the mixture injected subcutaneously into the right flank of their bodies. After 28 days following implantation, the mice were euthanized, and the specimen was taken for use in weight measures and in vitro experiments. Following the Canadian Council on Animal Care (CCAC) guideline, the tumor burden should not exceed 2000 mm^3^. Furthermore, when the body weight loss (BWL) of an individual mouse was 20%, that mouse was given a dosing holiday(s) until its body weight returned to baseline (BWL, 10%). This study was approved by the Ethics Committee of the Affiliated Taian City Central Hospital of Qingdao University (No.ATCCH2023710). All methods were carried out in accordance with relevant guidelines and regulations. The study was carried out in compliance with the ARRIVE guidelines.

### Microarray data source

The GEO2R online analyzer was used to conduct the analysis after GSE65372 was obtained from the GEO database. Using the GEO2R online analyzer, the next step was to identify DEGs and conduct a comparative study between tumor samples and non-tumor samples. Using the Benjamini–Hochberg technique, we deemed results significant if the adjusted P-value was less than 0.05. We conducted several assays after the gene expressions and survival data were obtained from TCGA datasets.

### Function enrichment

The clusterprofile R package was used to perform functional enrichment analysis of Gene Ontology (GO) and Kyoto Encyclopedia of Genes and Genomes (KEGG) pathway on DEGs. The results were assessed for functional significance. It was determined that the top findings were significant if the FDR was less than 0.05.

### The identification of novel diagnostic biomarkers in HCC

In order to discover relevant prognostic indicators, we used two separate machine learning approaches to create predictions about the course of the disease. The LASSO algorithm is a type of regression analysis that makes use of regularization in order to boost the accuracy of prediction. The "glmnet" package in R was utilized to carry out the LASSO regression approach in order to discover the genes that are substantially connected with the differentiation of HCC samples from normal samples. In LASSO, common model evaluation metrics were employed, such as cross-validation score, mean squared error, and R-squared value, to assess the fitting and predictive performance of the model. The support vector machine, sometimes known as SVM, is a popular supervised method of machine learning that may be used for both classification and regression. In SVM-RFE, the parameters of the support vector machine classifier were configured, such as the kernel function type and penalty coefficient. An RFE method was utilized in order to choose the most useful genes from the meta-data cohort in order to prevent overfitting.

### Tumor immune infiltration

Single-sample Gene Set Enrichment Analysis(ssGSEA) is a method for gene set enrichment analysis. It is a variant of Gene Set Enrichment Analysis (GSEA) designed to analyze gene expression data for individual samples or individuals, evaluating differences in gene set enrichment between different samples. Using the R package GSVA, the single sample gene set enrichment analysis (ssGSEA) algorithm was given a score based on 29 published immune-related genes. The scores were then standardized for each individual type of immune cell.

### Statistical analysis

Statistical analyses were performed using R 4.1.3 software packages. Student’s t-test and one-way ANOVA were respectively employed to evaluate two or multiple groups, for statistical significance. A p-value of less than 0.05 was considered statistically significant.

## Results

### Identification of DEGs in HCC

**Fig. 1:**
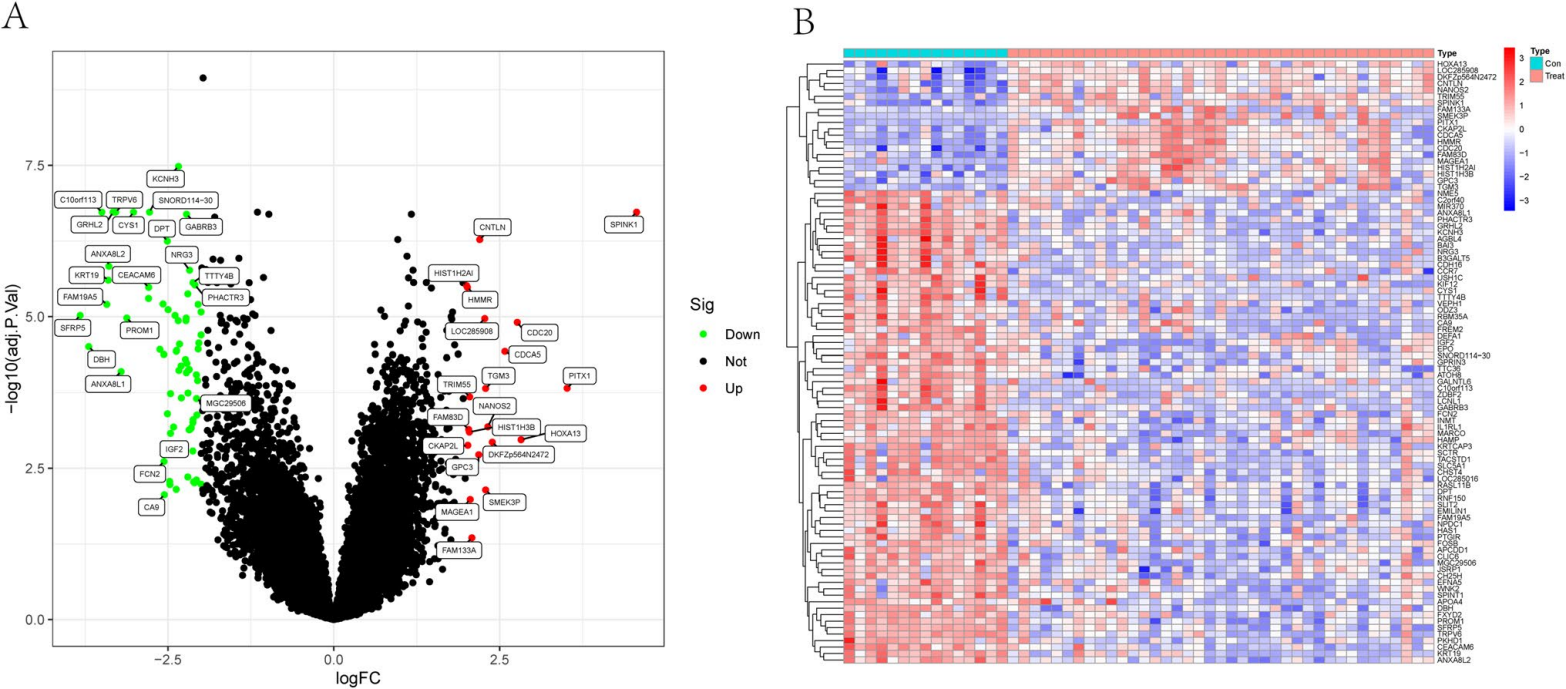
Differentially expressed genes between HCC specimens and normal specimens from GSE65372 datasets were shown in **A** Heat map and **B** Volcanic map

Firstly, a retrospective analysis was carried out on the collected data from GSE65372. A total of 103 DEGs were obtained: 20 genes were significantly upregulated and 73 genes were significantly downregulated (Fig. 1A, B).

### Functional correlation analysis

**Fig. 2:**
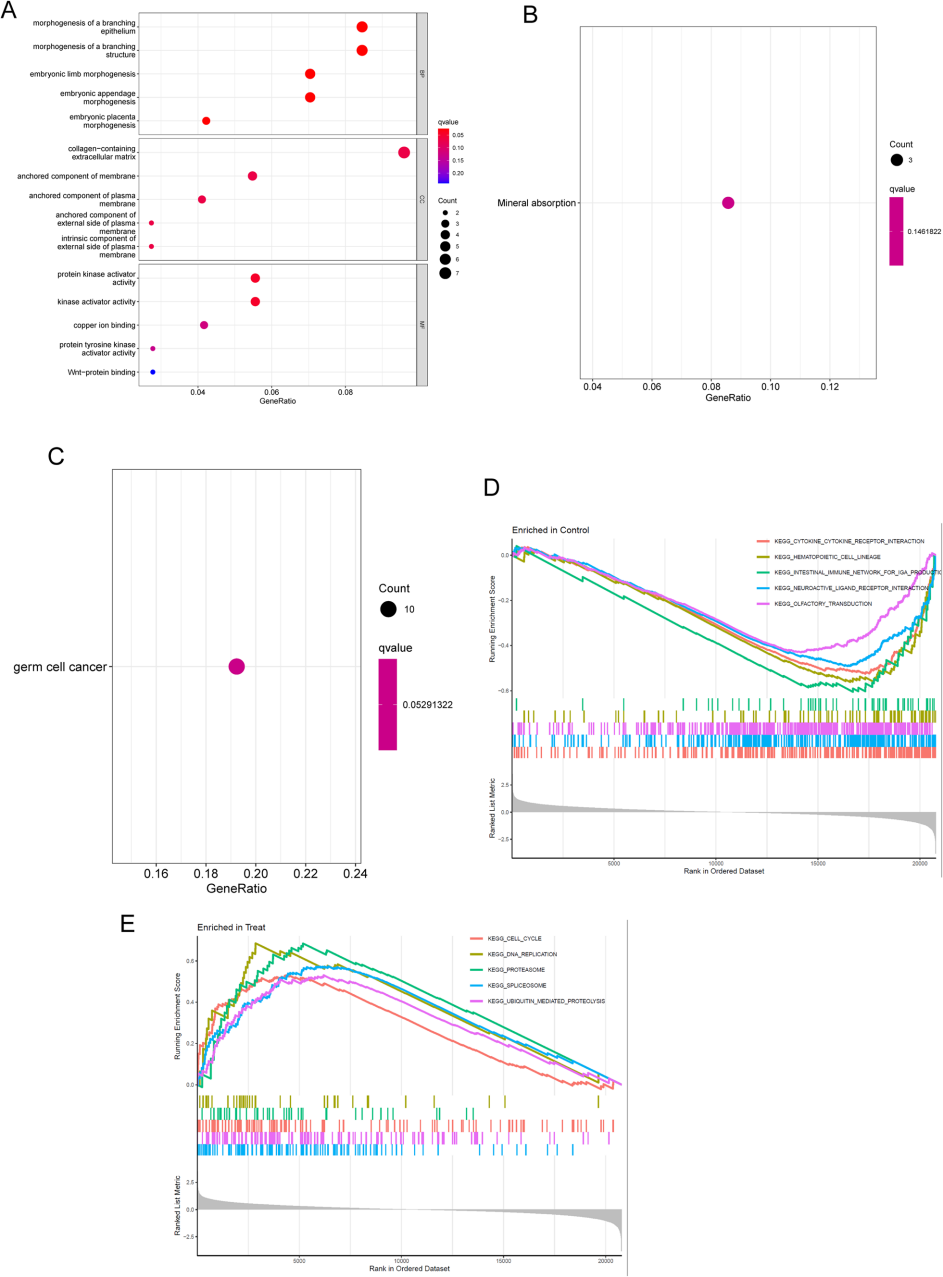
Functional enrichment analysis results. **A** GO analysis outcomes of DEGs, and the topmost 7 terms of every category were displayed. **B** The topmost 20 pathways of KEGG analysis. **C** Disease ontology enrichment analysis. **D** and **E** GESA analysis

To delve into the possible functions of 103 DEGs in HCC, we carried out GO analysis and observed that 103 DEGs were mainly associated with morphogenesis of a branching epithelium, morphogenesis of a branching structure, embryonic limb morphogenesis, collagen-containing extracellular matrix, anchored component of membrane, anchored component of plasma membrane, protein kinase activator activity, kinase activator activity and copper ion binding (Fig. 2A). The results of KEGG indicated that 103 DEGs were majorly enriched in Mineral absorption (Fig. 2B). In addition, DO assays revealed that 103 DEGs were related to germ cell cancer (Fig. 2C). Finally, we performed GSEA analysis and observed that KEGG_CYTOKINE_CYTOKINE_RECEPTOR_INTERACTION, KEGG_HEMATOPOIETIC_CELL_LINEAGE, EGG_INTESTINAL_IMMUNE_NETWORK_FOR_IGA_PRODUCTIO, KEGG_NEUROACTIVE_LIGAND_RECEPTOR_INTERACTION and KEGG_OLFACTORY_TRANSDUCTION were mainly enriched in control group (Fig. 2D) and KEGG_CELL_CYCLE, KEGG_DNA_REPLICATION, KEGG_PROTEASOME, KEGG_SPLICEOSOME and KEGG_UBIQUITIN_MEDIATED_PROTEOLYSIS were mainly enriched in treat group (Fig. 2E).

### Identification and validation of diagnostic genes

**Fig. 3:**
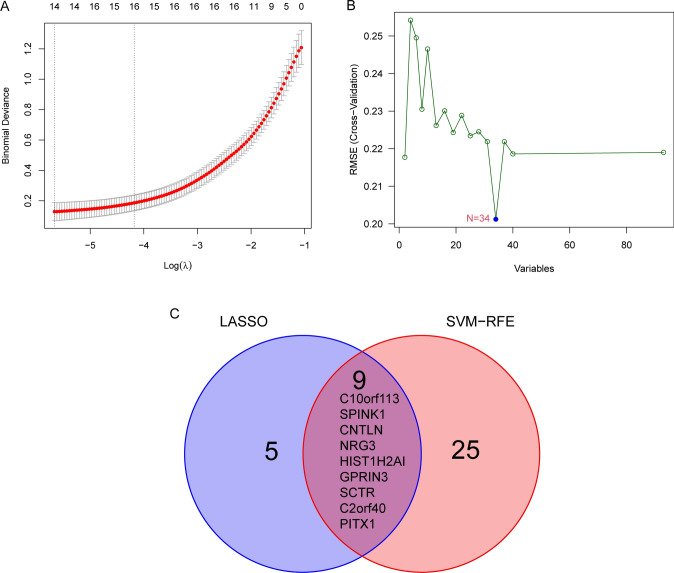
Machine learning for diagnostic genes for HCC patients. **A** Tuning feature selection in the LASSO model. **B** SVM-RFE algorithm. **C** Venn diagram showing nine critical genes(C10orf113, SPINK1, CNTLN, NRG3, HIST1H2AI, GPRIN3, SCTR, C2orf40 and PITX1) shared by SVM-RFE and LASSO assays

**Fig. 4:**
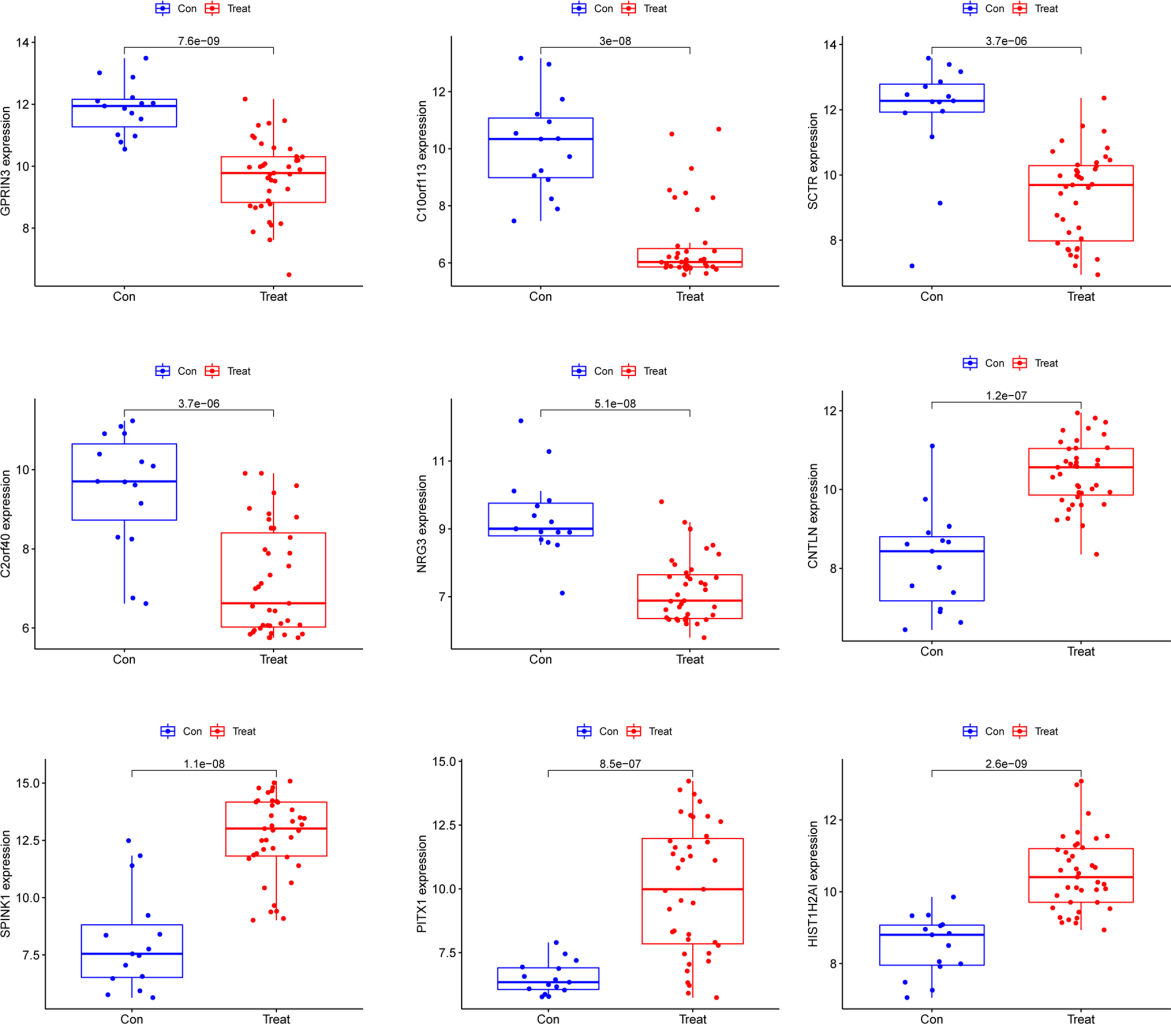
The expressing pattern of C10orf113, SPINK1, CNTLN, NRG3, HIST1H2AI, GPRIN3, SCTR, C2orf40 and PITX1 in HCC specimens and normal specimens via analyzing GSE65372 datasets

**Fig. 5:**
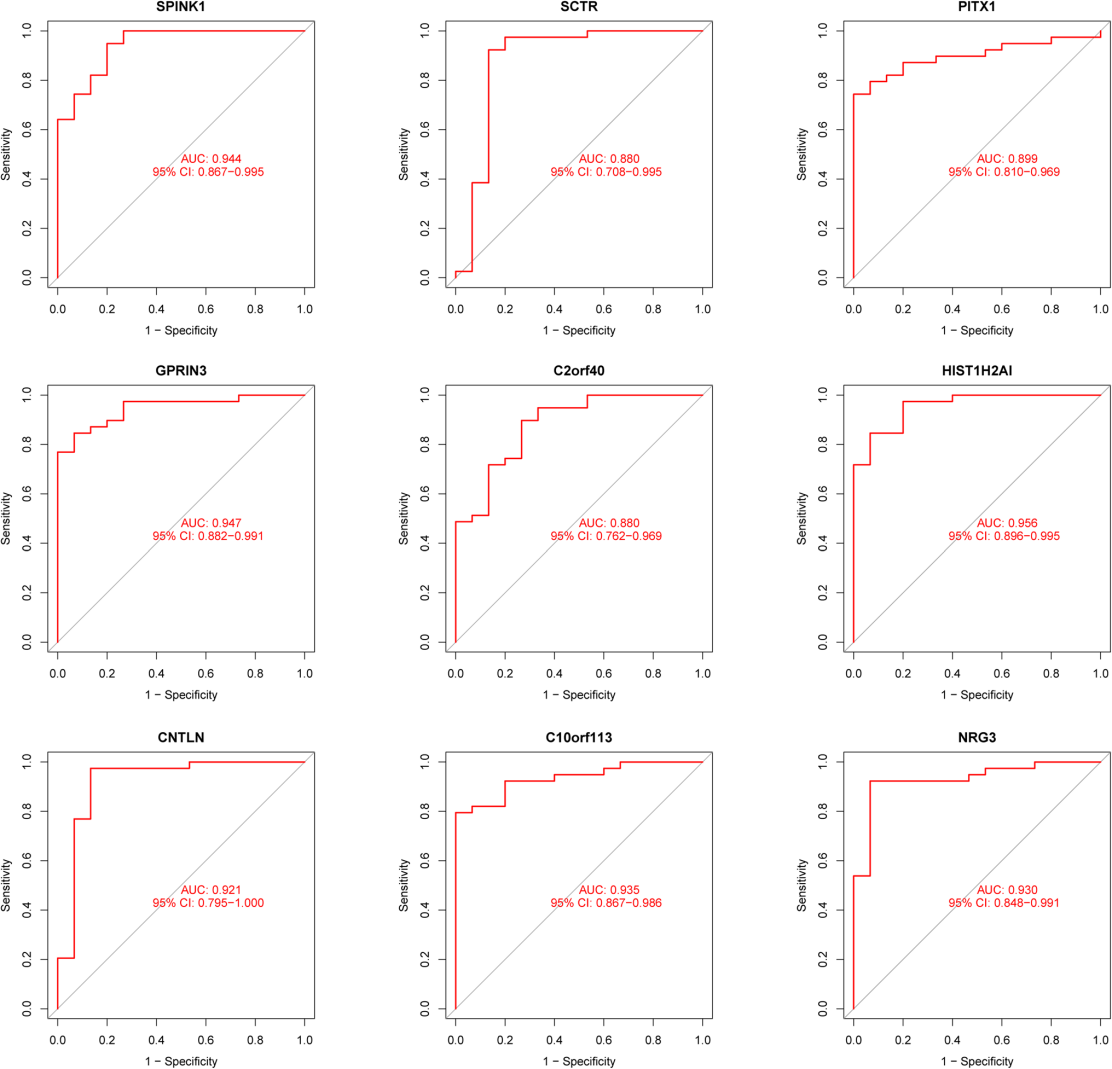
The diagnostic value of C10orf113, SPINK1, CNTLN, NRG3, HIST1H2AI, GPRIN3, SCTR, C2orf40 and PITX1 in screening HCC specimens from normal specimens using ROC assays

**Fig. 6:**
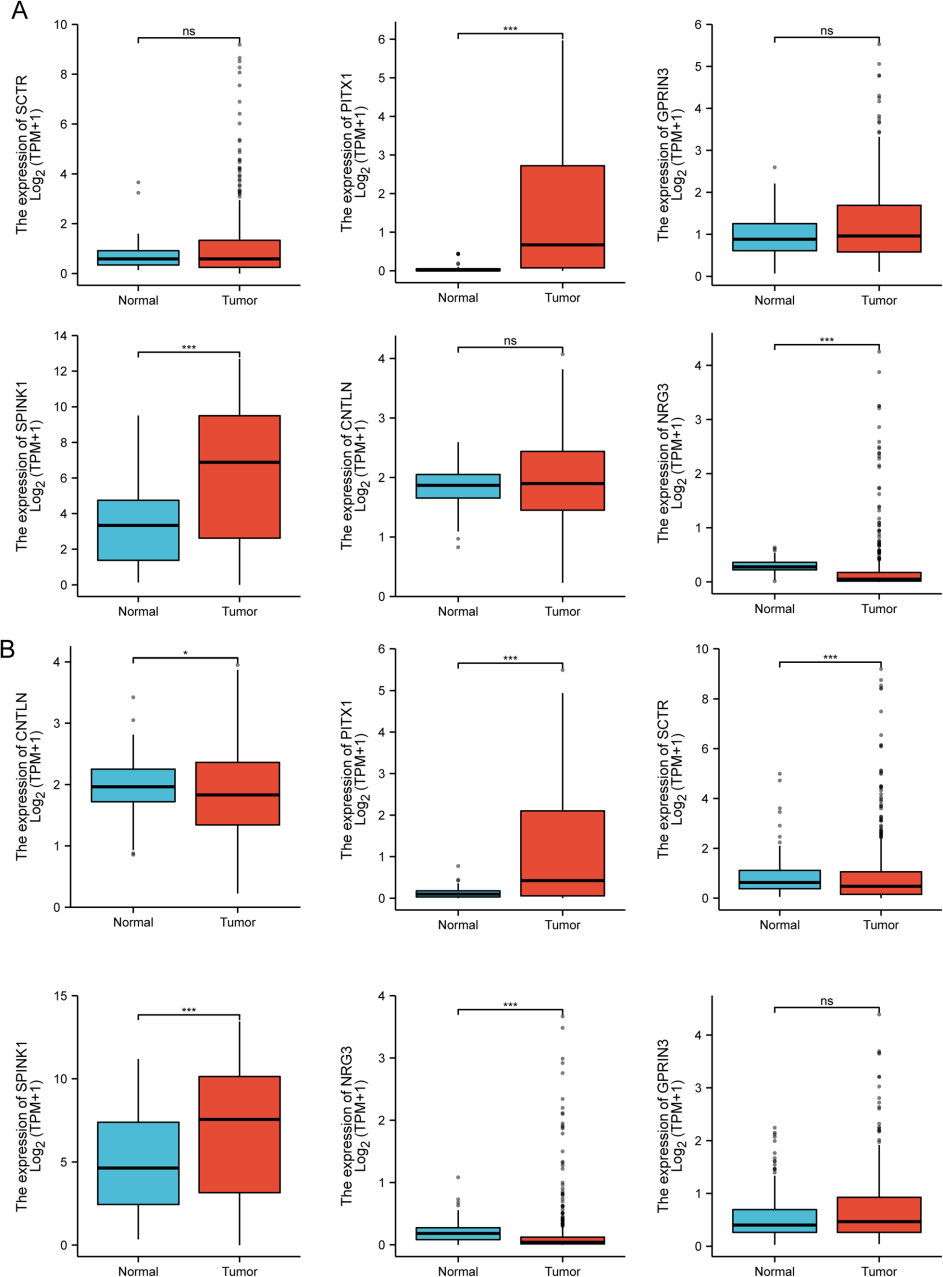
The expression of six diagnostic markers in **A** TCGA datasets and **B** TCGA and GTEx data

**Fig. 7:**
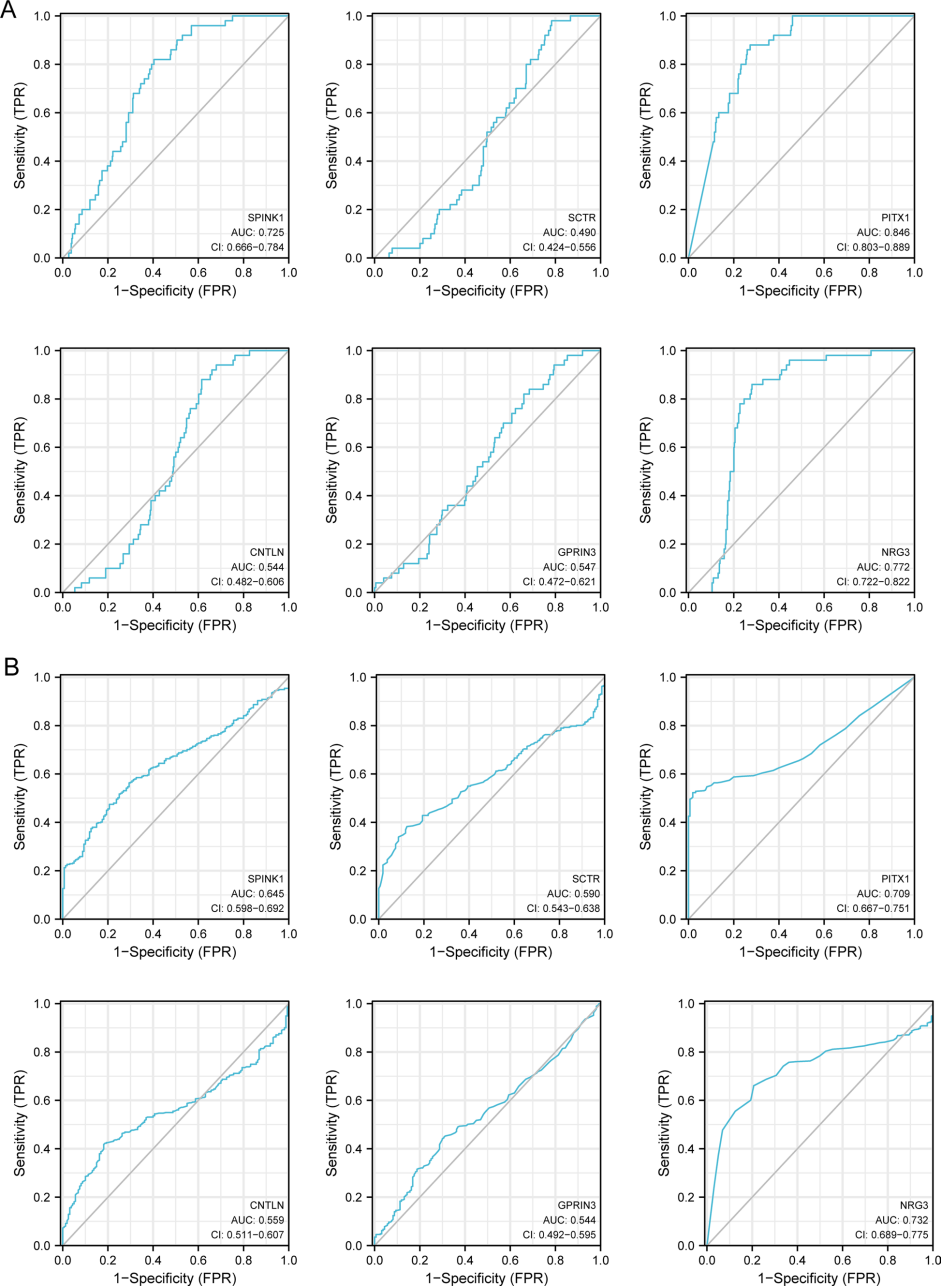
The diagnostic value of six diagnostic markers in **A** TCGA datasets and **B** TCGA and GTEx data

In order to conduct a search for potentially useful biomarkers, two separate algorithms were used. The DEGs were narrowed down by the use of the LASSO regression method, and the end result was the identification of 14 variables as potential diagnostic biomarkers for HCC (Fig. 3A). The SVM-RFE method was utilized so that the properties of the DEGs could be whittled down to a chosen group of five criteria (Fig. 3B). The nine overlapping genes (C10orf113, SPINK1, CNTLN, NRG3, HIST1H2AI, GPRIN3, SCTR, C2orf40 and PITX1) were obtained (Fig. 3C). The expressing pattern of the nine critical genes were shown in Fig. 4. In addition, the results of ROC assays confirmed the diagnostic value of all nine critical genes in screening HCC specimens from normal specimens with an AUC > 0.7 (Fig. 5). On the other hand, we further analyzed the expression of C10orf113, SPINK1, CNTLN, NRG3, HIST1H2AI, GPRIN3, SCTR, C2orf40 and PITX1 using TCGA datasets and GTEx data. As shown in Fig. 6A, B, we discovered that the expression of PITX1 and SPINK1 was significantly higher in HCC specimens compared with normal specimens in both the TCGA and GTEx data. This was the case regardless of the kind of specimen. ROC tests were also used, which provided further confirmation of PITX1 and SPINK1's diagnostic usefulness (Fig. 7A, B).

### The prognostic value of the critical genes using TCGA datasets

**Fig. 8:**
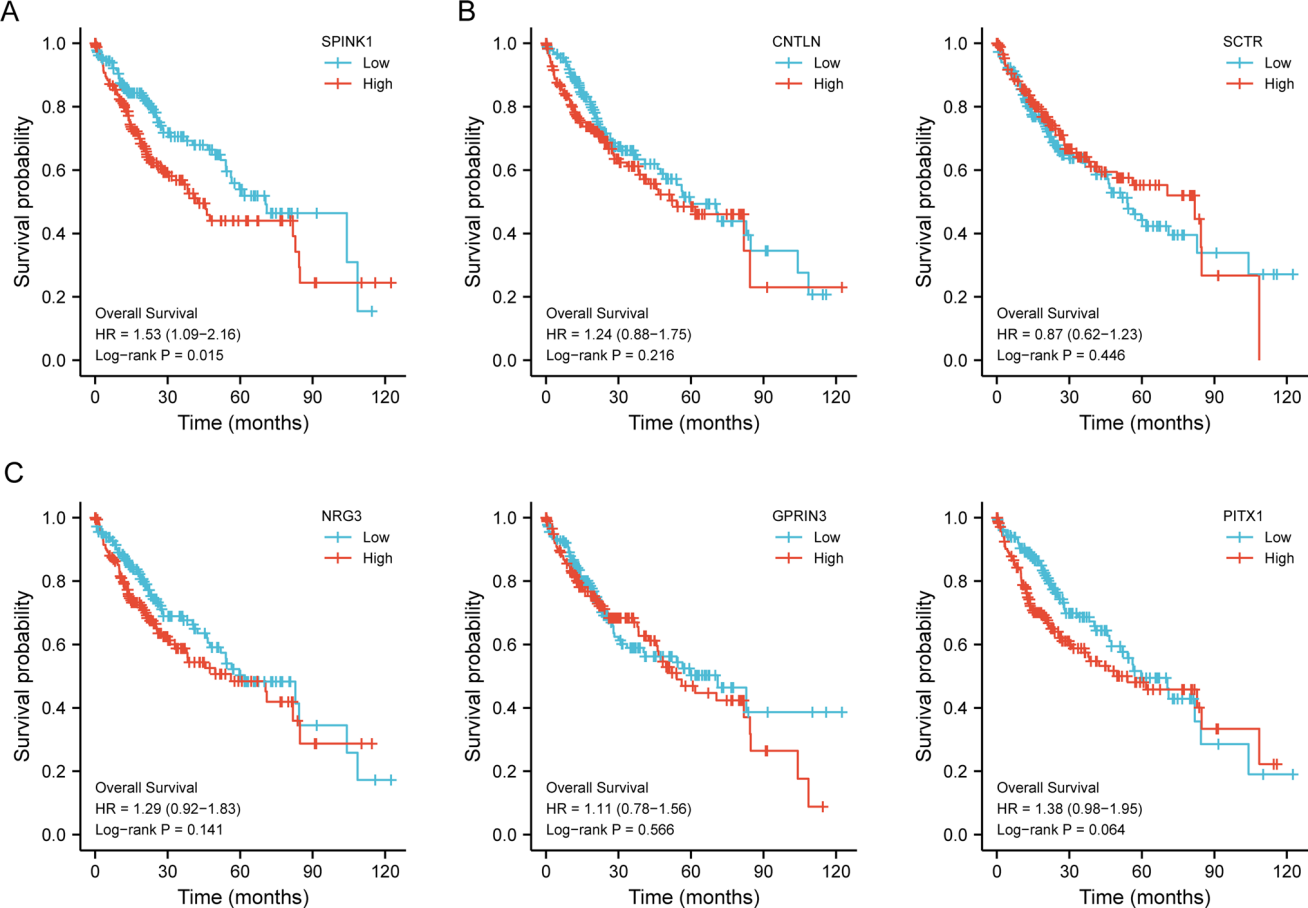
The prognostic value of six diagnostic markers in HCC from TCGA datasets. **A** High SPINK1 expression was related to poor prognosis of HCC patients. **B** and **D** Kaplan–Meier curves showed the associations between SPINK1 expressions and other five diagnostic genes

After that, we conducted another research utilizing TCGA datasets to investigate the predictive significance of six important genes in HCC patients. We only found that patients with high SPINK1 expression had a lower overall survival than those with low SPINK1 expression (Fig. 8A, p = 0.015). The other five genes(CNTLN, SCTR, NRG3, GPRIN3 and PITX1) were not associated with the clinical outcome of HCC patients (Fig. 8B and C). In addition, to examine the correlations between SPINK1 expressions and clinicopathological characteristics, all 374 HCC patients were divided into two subgroups according to mean value: a high-SPINK1 group (187 cases) and a low-SPINK1 group (187 cases). As shown in Table 1, we found that high expression of SPINK1 was associated with advanced Histologic grade. Finally, we further performed multivariate Cox regression analysis to evaluate the prognostic value of SPINK1 in HCC patients. As shown in Table 2, we found that SPINK1 was an independent prognostic factor for overall survival of HCC patients (HR = 1.589, 95% CI 1.098–2.301, p = 0.014).

**Table 1:** Correlation of clinicopathological features of HCC with SPINK1 expression levels

Characteristic	Low expression of SPINK1	High expression of SPINK1	p
n	187	187	
Gender, n (%)			0.825
Female	62 (16.6%)	59 (15.8%)	
Male	125 (33.4%)	128 (34.2%)	
Age, n (%)			0.502
< = 60	85 (22.8%)	92 (24.7%)	
> 60	102 (27.3%)	94 (25.2%)	
Pathologic stage, n (%)			0.626
Stage I	89 (25.4%)	84 (24%)	
Stage II	43 (12.3%)	44 (12.6%)	
Stage III	41 (11.7%)	44 (12.6%)	
Stage IV	4 (1.1%)	1 (0.3%)	
Histologic grade, n (%)			0.031
G1	33 (8.9%)	22 (6%)	
G2	92 (24.9%)	86 (23.3%)	
G3	56 (15.2%)	68 (18.4%)	
G4	2 (0.5%)	10 (2.7%)	
Age, median (IQR)	62 (51.5, 69)	61 (52, 68)	0.732

**Table 2:** Univariate and multivariate analyses for overall survival in HCC patients

Characteristics	Total (N)	Univariate analysis		Multivariate analysis	
		Hazard ratio (95% CI)	P value	Hazard ratio (95% CI)	P value
Gender	373				
Female	121	Reference			
Male	252	0.793 (0.557–1.130)	0.200		
Age	373				
< = 60	177	Reference			
> 60	196	1.205 (0.850–1.708)	0.295		
Histologic grade	368				
G1&G2	233	Reference			
G3&G4	135	1.091 (0.761–1.564)	0.636		
Pathologic stage	349				
Stage I & Stage II	259	Reference			
Stage III & Stage IV	90	2.504 (1.727–3.631)	** < 0.001**	2.534 (1.747–3.676)	** < 0.001**
SPINK1	373				
Low	187	Reference			
High	186	1.539 (1.086–2.181)	**0.015**	1.589 (1.098–2.301)	**0.014**

### SPINK1 expression correlates to immune infiltration

**Fig. 9:**
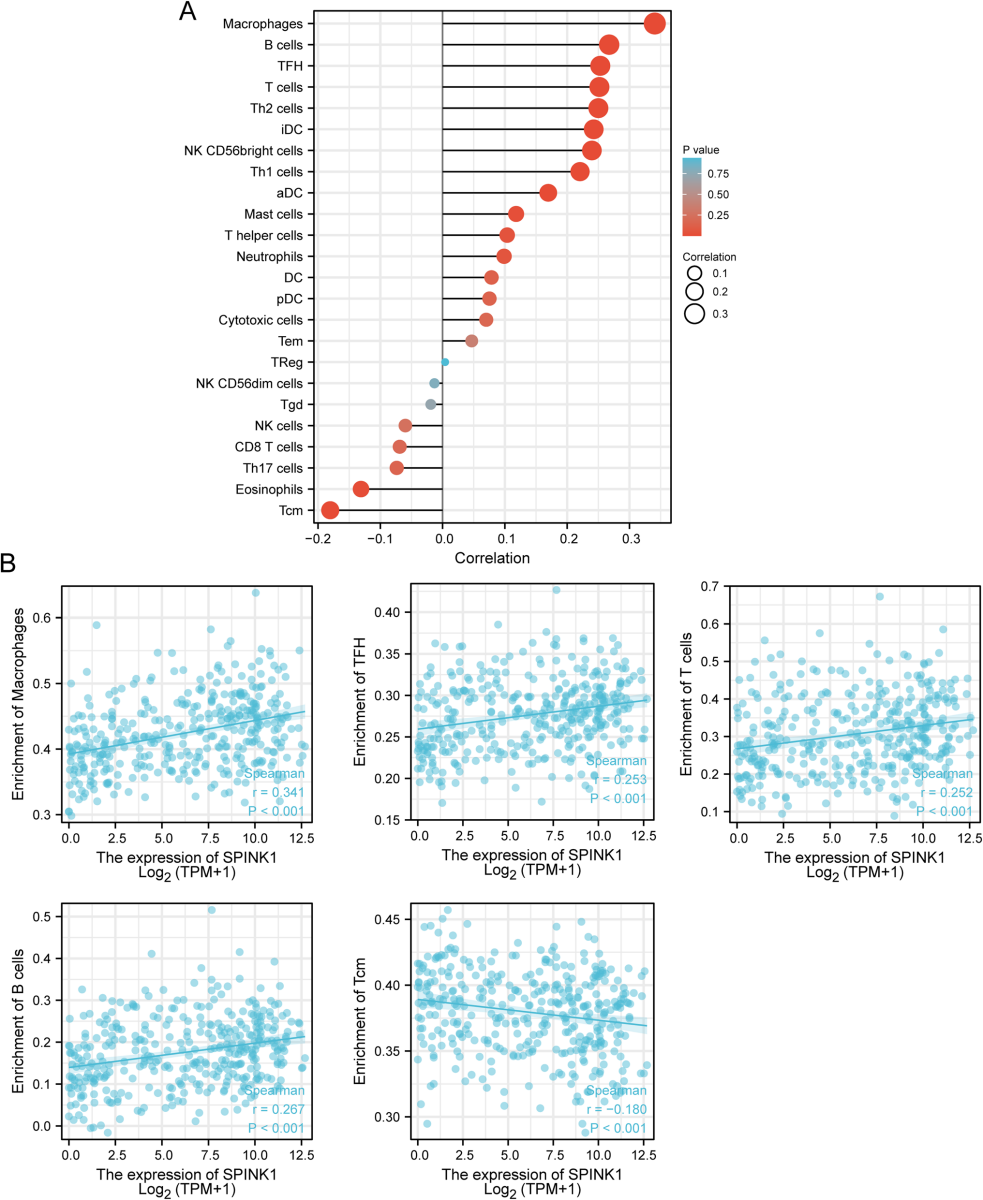
**A** and **B** SPINK1 level was linked to the immune infiltration in the TME

The relevance between SPINK1 expression and the ssGSEA-quantified level of immune cell infiltration was processed by spearman correlation. SPINK1 level was positively associated with Macrophages, B cells, TFH, T cells, Th2 cells, iDC, NK CD56bright cells, Th1 cells, aDC, while negatively associated with Tcm and Eosinophils (Fig. 9A, B).

### *The expression of SPINK1 in our cohort and its oncogenic roles in HCC *via* regulating β-catenin*

**Fig. 10:**
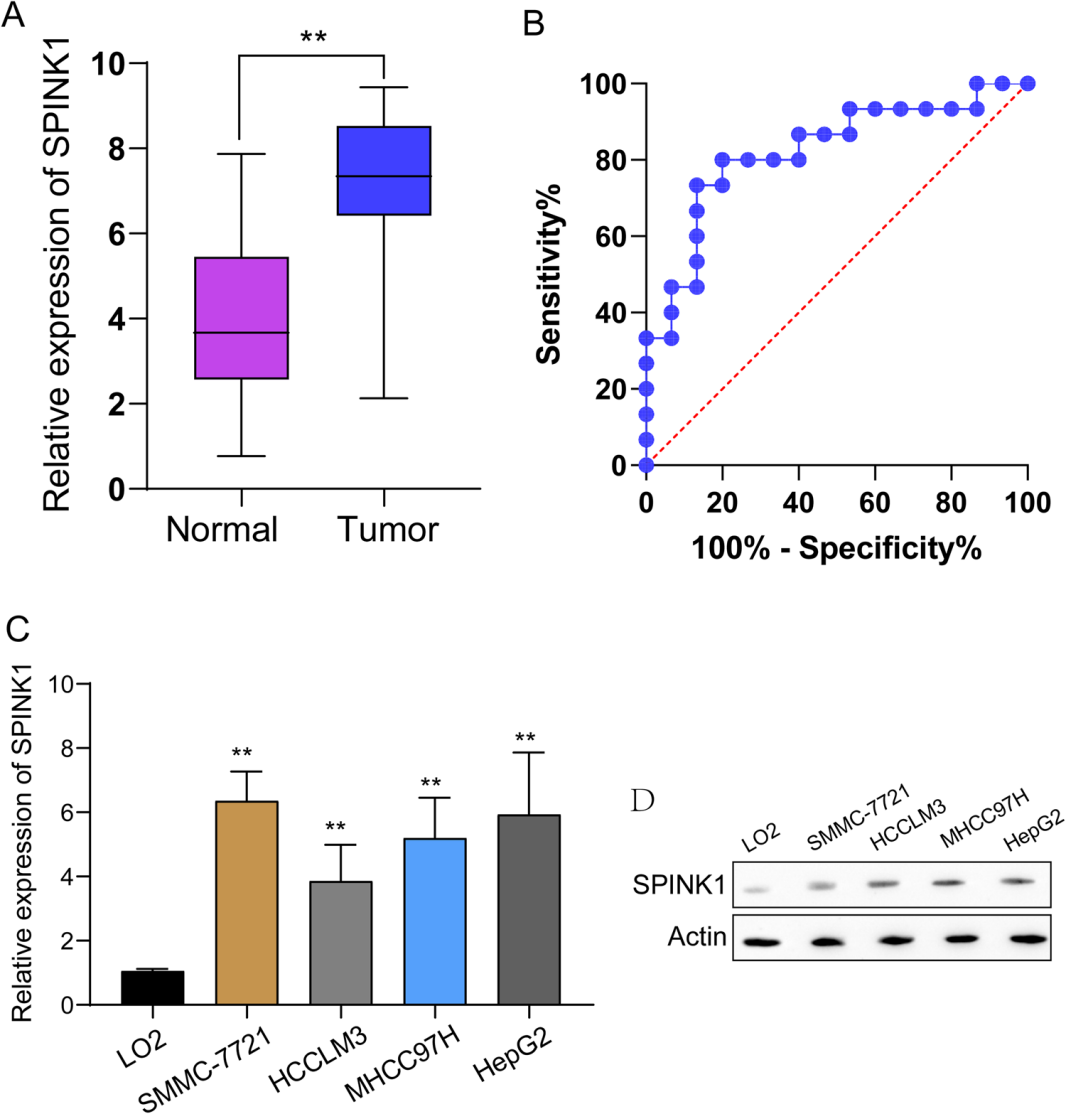
SPINK1 was upregulated in HCC specimens and cells. **A** RT-PCR assats determined SPINK1 expressions in 15 paired HCC samples and normal specimens. **B** ROC assays for the diagnostic values of SPINK1 expressions in screening HCC specimens from normal specimens. **C** and **D** SPINK1 expressions in HCC cells were examined by RT-PCR and Western blot

**Fig. 11:**
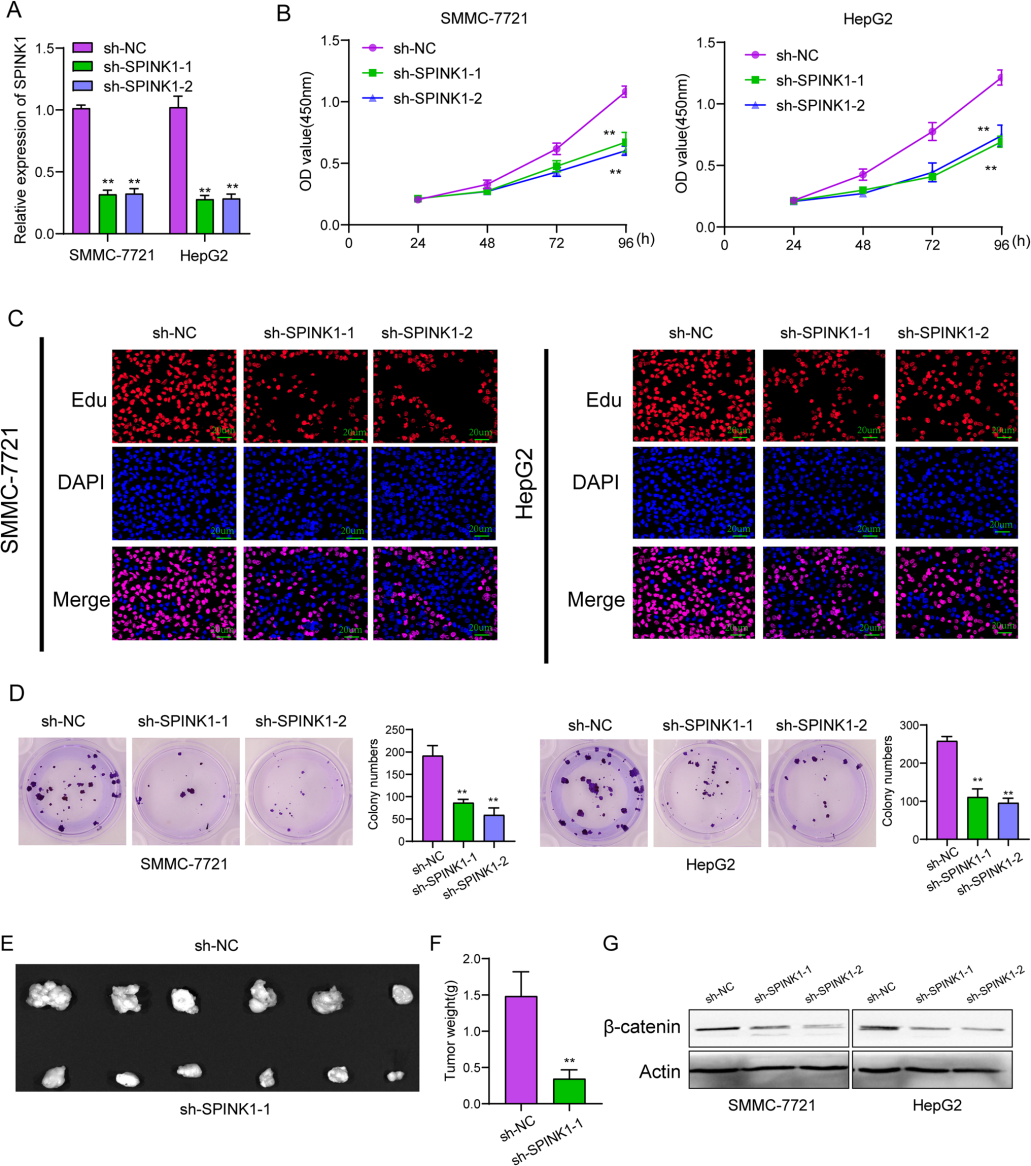
SPINK1 depletion suppressed cell growth via regulating Wnt/β-catenin signaling. **A** RT-PCR for the down-regulation of SPINK1 in SMMC-7721 and HepG2 cells after the transfection of sh-SPINK1-1 or sh-SPINK1-2. **B** Assays with CCK-8 were used to measure the rate of proliferation of HCC cells following infection. **C** EdU assays examined the proliferative cells. **D** Colony formation assays. **E** Through the injection of SPINK1-knocked down HepG2 cells into nude mice, a Xenograft tumor model was created. **F** On day 28 following the injection, the weight of the tumor was measured after the model mice had been sacrificed under anaesthetic. **G** Western blot for the expressions of β-catenin in SMMC-7721 and HepG2 cells after the transfection of sh-SPINK1-1 or sh-SPINK1-2

In order to provide evidence that SPINK1 is expressed in HCC, we carried out RT-PCR analysis on a total of 15 HCC tissue samples and 15 noncancerous tissue samples that were matched. As compared with non-tumor specimens, we discovered that the level of expression of SPINK1 was much higher in HCC samples (Fig. 10A). In addition, ROC experiments validated the diagnostic significance of SPINK1 expression in distinguishing HCC specimens from non-tumor specimens with an area under the curve (AUC) greater than 0.70 (Fig. 10B). Moreover, we examined the expression of SPINK1 in several HCC cell lines and found that SPINK1 expression was highly expressed in four HCC cell lines compared with LO2 cells (Fig. 10C and Fig. 10D). The next step that we took was to investigate the role that SPINK1 played in the progression of malignancy in HCC cells. The results of qPCR studies showed that SPINK1 shRNA transfection resulted to SPINK1 knockdown in HCC cells with an efficiency of over 65% (Fig. 11A). After that, the outcomes of the CCK-8 tests indicated that the measurement of OD 450 nm absorbance values verified that the proliferation rates of SPINK1 depleted cells displayed a considerable decline in comparison to that of the control cells (Fig. 11B). The results of the EdU staining further validated the findings about the proliferation. The findings demonstrated that lowering levels of SPINK1 led to a significant decrease in the number of HCC cells that were actively dividing (Fig. 11C). In addition, clonogenic experiments revealed that the capabilities of clone production were diminished after SPINK1 was removed from the cells (Fig. 11D). Finally, we performed vivo experiments and confirmed that knockdown of SPINK1 distinctly suppressed tumor growth in HCC (Fig. 11E, F). HCC is characterized by an abnormal stimulation of the Wnt/catenin signaling system, which plays a role in the growth of tumors. Then, we performed western blot and found that knockdown of SPINK1 distinctly suppressed the expression of β‐catenin in SMMC-7721 and HepG2 cells (Fig. 11G).

## Discussion

In recent years, significant advancements have been made in HCC research, particularly in the identification and utilization of tumor markers to facilitate early detection and diagnosis [27, 28]. The development is particularly noteworthy in light of the fact that HCC is becoming increasingly common [29]. Cancer cells secrete chemicals called tumor markers, which can be found in the blood or other physiological fluids and can be used to diagnose cancer. They can assist in determining whether or not cancer is present and tracking its development. Several tumor markers have been identified for HCC, including alpha-fetoprotein, des-gamma-carboxy prothrombin, and glypican-3 [30, 31]. Studies have shown that measuring these markers, along with imaging tests, can improve the accuracy of HCC diagnosis and help determine the most effective treatment options. Additionally, ongoing research is focused on the discovery of new tumor markers and the development of more advanced diagnostic and treatment approaches for HCC. Although HCC markers play an important role in the early diagnosis and treatment of HCC, there are also some shortcomings with the currently known markers. Firstly, some HCC patients may experience false negative or false positive results due to various factors such as individual differences, underlying health conditions or medication usage. This can lead to delayed diagnosis or unnecessary treatments, which can cause physical and emotional distress to patients. Secondly, the sensitivity and specificity of existing markers are not ideal [32]. For example, AFP has been widely used as a marker for HCC, but its sensitivity and specificity are not high enough to be relied on as the only diagnostic tool. Additionally, the limited number of known markers means that they cannot fully represent the heterogeneity of HCC, which may make it difficult to detect some types of HCC. Hence, additional research is essential to unearth new markers and enhance the sensitivity and specificity of current markers, aiming to offer improved guidance for the diagnosis and treatment of HCC.

Machine learning has been increasingly utilized in the identification of tumor markers, which are molecules or substances that indicate the presence of a tumor in the body [33, 34]. By analyzing large amounts of data, including genetic and clinical information, machine learning algorithms can identify patterns and correlations that are indicative of certain tumor markers. This can aid in the early detection and diagnosis of cancer, as well as in the development of personalized treatment plans. Additionally, machine learning can help identify new and previously unknown tumor markers, leading to advances in cancer research and treatment. In this study, we analyzed GSE65372 datasets and identified 103 dysregulated genes between HCC specimens and normal specimens. Function Enrichment assays suggested the 103 genes were involved in tumor progression. Then, we performed LASSO and SVM-RFE algorithm, identifying nine critical diagnostic genes, including C10orf113, SPINK1, CNTLN, NRG3, HIST1H2AI, GPRIN3, SCTR, C2orf40 and PITX1. However, in TCGA datasets, only PITX1 and SPINK1 were confirmed to show a strong diagnostic value in screening HCC specimens from normal specimens. In addition, we discovered that SPINK1 was the sole gene that was related with the clinical outcome of HCC patients. It is important to note that a multivariate Cox regression analysis indicated that SPINK1 was an independent predictive factor for the overall survival of HCC patients. Kazal et al. [35] were the ones who made the initial discovery of the Serine peptidase inhibitor Kazal type I (SPINK1) in bovine pancreas extracts. The researchers characterized the molecule as a pancreatic secretory trypsin inhibitor. Another study group extracted SPINK1 from the urine of ovarian cancer patients and further characterized its properties. Further research determined that SPINK1 and TATI are two names for the same molecule [36]. Prior research has suggested a link between mutations in the gene SPINK1 and an increased likelihood of developing pancreatic cancer, particularly in individuals who suffered from chronic pancreatitis [37]. Pyrosequencing found that individuals who suffered from chronic pancreatitis due to a mutation in the SPINK1 gene called c.101A > G (p. N34S) had a 12-fold greater likelihood of getting pancreatic cancer in comparison to controls. Many studies have been conducted to investigate the predictive value of SPINK1 in cancer. It has been determined that SPINK1 functions as a prognostic marker for lung cancer. Patients with lung adenocarcinoma who had greater SPINK1 levels were linked to a less favorable overall survivals [38]. It was also reported in a cohort of 273 instances of hepatocellular carcinoma that there was a relationship with having a poor overall survival. It was shown that the non-serous histological tumor subtypes of ovarian cancer, such as endometrioid, clear cell, and mucinous subtypes, had a connection with low survival rates. These subtypes include endometrioid tumors. Throughout the course of the inquiry, this came to light [39]. In patients with muscle-invasive bladder cancer, SPINK1 is also regarded to be one of nine critical biomarkers that can predict overall survival. In addition, it has been shown that SPINK1 promotes the development and survival of tumor cells in prostate cancer by suppressing the activity of specific enzymes that are involved in apoptosis, also known as programmed cell death. This is done in the process of preventing apoptosis [40]. Overexpression of the protein SPINK1 has been linked to enhanced invasiveness and metastasis in pancreatic cancer, presumably as a result of the activation of signaling pathways that drive cell motility and invasion. Previously, Huang et al. reported that the expression of SPINK1 was considerably higher in the tumor tissues as compared to the corresponding para-tumor tissues. A substantial correlation was found between higher SPINK1 expression in the tumor and the production of portal vein tumor thrombus as well as a shorter overall survival time. The amount of SPINK1 that was expressed in tumor tissue served as an independent predictor of overall survival. SPINK1 induced an increase in HCC cell lines' capacity for proliferation, as well as accelerated migration and invasion [41]. Our findings were consistent with previous findings. However, this study firstly used machine learning and GEO and TCGA datasets to prove the diagnostic and prognostic value of SPINK1 in HCC.

HCC is a complex disease that involves various cellular and molecular factors in its development and progression. The immune microenvironment of the liver plays a critical role in regulating the balance between tumor-promoting and tumor-suppressing activities [42, 43]. In patients with HCC, the immune system is often impaired, which leads to the accumulation of immune suppressive cells and molecules within the tumor microenvironment. Certain immune cells, such as regulatory T cells and myeloid-derived suppressor cells, inhibit the body's natural anti-tumor response, which in turn encourages the expansion and continued survival of cancer cells [44, 45]. Furthermore, the immune microenvironment of the liver is impacted by various environmental factors, including chronic viral infections, alcohol consumption, and obesity, which further amplify immune dysfunction and elevate the risk of HCC development [44, 46]. Therefore, understanding the complex interplay between the immune microenvironment and HCC is critical for the development of effective immunotherapeutic approaches for the treatment of this disease. Immunotherapy has steadily developed into a potentially beneficial therapeutic option for advanced HCC. It was very recently discovered that the amounts of immune infiltration can alter how well immunotherapy works. On the other hand, there is a paucity of information about the relationship between SPINK1 and immunological infiltrates, as far as we are aware. To determine whether or not the expressions of SPINK1 were related to the immune microenvironment of HCC, we accessed the TCGA database and downloaded the data of 374 cancer patients who were still alive at the time of the study. Our analysis showed that SPINK1 level was positively associated with Macrophages, B cells, TFH, T cells, Th2 cells, iDC, NK CD56bright cells, Th1 cells, aDC, while negatively associated with Tcm and Eosinophils. Our findings suggested SPINK1 may influence HCC progression via regulating immune-related mechanisms.

The Wnt/β-catenin signaling pathway is intimately connected to HCC and is an essential system that plays a role in the control of cell proliferation, differentiation, and survival throughout embryonic development as well as in adult tissue development [47]. However, when this pathway is aberrantly activated, it can promote the progression of tumors, including HCC [48]. Under typical conditions, activation of the Wnt/β-catenin signaling pathway is dependent on the binding of extracellular Wnt proteins to cell surface receptors, which in turn starts the signal transduction process [49]. This leads to the accumulation of β-catenin protein in the cytoplasm, which then translocates to the nucleus and binds to transcription factors, promoting the transcription of genes involved in cell proliferation and growth. In HCC, the Wnt/β-catenin signaling pathway is often disrupted, which can result from various mechanisms, including mutations in Wnt receptors, abnormal expression of intracellular signaling molecules, and the loss of tumor suppressor genes. Inappropriate stimulation of the Wnt/β-catenin signaling pathway results in inappropriate accumulation of β-catenin and excessive transcriptional activity, which in turn promotes HCC cell proliferation, anti-apoptosis, and invasive capacities [50, 51]. Furthermore, the Wnt/β-catenin signaling pathway can interact with other pathways involved in HCC, such as the PI3K/AKT and Ras/MAPK signaling pathways, further enhancing the development and progression of HCC [52, 53]. In this study, we confirmed that knockdown of SPINK1 distinctly suppressed the expression of β-catenin, suggesting that SPINK1 promoted the proliferation of HCC cells via regulating Wnt/β-catenin signaling.

## Conclusion

Based on our research findings, we observed elevated SPINK1 expressions in HCC tissues when compared to non-tumor specimens. Furthermore, higher SPINK1 levels were correlated with increased histological grades and poorer overall survival among HCC patients. Additionally, our experiments verified that the downregulation of SPINK1 inhibited the activity of the Wnt/β-catenin signaling pathway. Our research results indicate that SPINK1 holds the potential to not only serve as a diagnostic and prognostic marker but also as a promising target for immunotherapy in HCC.

## Data Availability

The datasets generated during and/or analyzed during the current study are available from the corresponding author on reasonable request.

## References

[1] Forner A, Reig M, Bruix J (2018). Hepatocellular carcinoma. Lancet.

[2] Lee TK, Guan XY, Ma S (2022). Cancer stem cells in hepatocellular carcinoma - from origin to clinical implications. Nat Rev Gastroenterol Hepatol.

[3] Chidambaranathan-Reghupaty S, Fisher PB, Sarkar D (2021). Hepatocellular carcinoma (HCC): epidemiology, etiology and molecular classification. Adv Cancer Res.

[4] Yang S, Wang J, Wang S, Zhou A, Zhao G, Li P (2022). Roles of small extracellular vesicles in the development, diagnosis and possible treatment strategies for hepatocellular carcinoma (Review). Int J Oncol.

[5] Liccioni A, Reig M, Bruix J (2014). Treatment of hepatocellular carcinoma. Digest Dis.

[6] Ganesan P, Kulik LM (2023). Hepatocellular carcinoma: new developments. Clin Liver Dis.

[7] Cho E, Cho HA, Jun CH, Kim HJ, Cho SB, Choi SK (2019). A review of hepatocellular carcinoma in elderly patients focused on management and outcomes. In vivo.

[8] Testino G, Leone S, Patussi V, Scafato E, Borro P (2016). Hepatocellular carcinoma: diagnosis and proposal of treatment. Minerva Med.

[9] Greener JG, Kandathil SM, Moffat L, Jones DT (2022). A guide to machine learning for biologists. Nat Rev Mol Cell Biol.

[10] Peiffer-Smadja N, Rawson TM, Ahmad R, Buchard A, Georgiou P, Lescure FX, Birgand G, Holmes AH (2020). Machine learning for clinical decision support in infectious diseases: a narrative review of current applications. Clin Microbiol Infect.

[11] Gupta R, Srivastava D, Sahu M, Tiwari S, Ambasta RK, Kumar P (2021). Artificial intelligence to deep learning: machine intelligence approach for drug discovery. Mol Diversity.

[12] Choi RY, Coyner AS, Kalpathy-Cramer J, Chiang MF, Campbell JP (2020). Introduction to machine learning, neural networks, and deep learning. Transl Vision Sci Technol.

[13] Handelman GS, Kok HK, Chandra RV, Razavi AH, Lee MJ, Asadi H (2018). eDoctor: machine learning and the future of medicine. J Intern Med.

[14] Triantafyllidis AK, Tsanas A (2019). Applications of machine learning in real-life digital health interventions: review of the literature. J Med Internet Res.

[15] Silva GFS, Fagundes TP, Teixeira BC, Chiavegatto Filho ADP (2022). Machine learning for hypertension prediction: a systematic review. Curr Hypertens Rep.

[16] Aafjes-van Doorn K, Kamsteeg C, Bate J, Aafjes M (2021). A scoping review of machine learning in psychotherapy research. Psychother Res.

[17] Pearce EL, Pearce EJ (2013). Metabolic pathways in immune cell activation and quiescence. Immunity.

[18] Marzagalli M, Ebelt ND, Manuel ER (2019). Unraveling the crosstalk between melanoma and immune cells in the tumor microenvironment. Semin Cancer Biol.

[19] Sadighi Akha AA (2018). Aging and the immune system: an overview. J Immunol Methods.

[20] Schreiber RD, Old LJ, Smyth MJ (2011). Cancer immunoediting: integrating immunity’s roles in cancer suppression and promotion. Science.

[21] Gardner A, Ruffell B (2016). Dendritic cells and cancer immunity. Trends Immunol.

[22] Locy H, de Mey S, de Mey W, De Ridder M, Thielemans K, Maenhout SK (2018). Immunomodulation of the tumor microenvironment: turn foe into friend. Front Immunol.

[23] Bommareddy PK, Shettigar M, Kaufman HL (2018). Integrating oncolytic viruses in combination cancer immunotherapy. Nat Rev Immunol.

[24] Riera-Domingo C, Audigé A, Granja S, Cheng WC, Ho PC, Baltazar F, Stockmann C, Mazzone M (2020). Immunity, hypoxia, and metabolism-the ménage à trois of cancer: implications for immunotherapy. Physiol Rev.

[25] Marciscano AE, Anandasabapathy N (2021). The role of dendritic cells in cancer and anti-tumor immunity. Semin Immunol.

[26] Yu MW, Quail DF (2021). Immunotherapy for glioblastoma: current progress and challenges. Front Immunol.

[27] Kim DW, Talati C, Kim R (2017). Hepatocellular carcinoma (HCC): beyond sorafenib-chemotherapy. J Gastrointestinal Oncol.

[28] Wang W, Wei C (2020). Advances in the early diagnosis of hepatocellular carcinoma. Genes Dis.

[29] Jiang Y, Han Q, Zhao H, Zhang J (2021). The mechanisms of HBV-induced hepatocellular carcinoma. J Hepatocell Carcinoma.

[30] Gentile D, Donadon M, Lleo A, Aghemo A, Roncalli M, di Tommaso L, Torzilli G (2020). Surgical treatment of hepatocholangiocarcinoma: a systematic review. Liver Cancer.

[31] Mehta N, Bhangui P, Yao FY, Mazzaferro V, Toso C, Akamatsu N, Durand F, Ijzermans J, Polak W, Zheng S *et al*: Liver Transplantation for Hepatocellular Carcinoma. Working Group Report from the ILTS Transplant Oncology Consensus Conference. *Transplantation.* 2020, **104**(6): 1136–1142. 10.1097/TP.0000000000003174 32217938

[32] De Stefano F, Chacon E, Turcios L, Marti F, Gedaly R (2018). Novel biomarkers in hepatocellular carcinoma. Digest Liver Dis.

[33] Huang S, Cai N, Pacheco PP, Narrandes S, Wang Y, Xu W (2018). Applications of support vector machine (SVM) learning in cancer genomics. Cancer Genom Proteom.

[34] Kong J, Ha D, Lee J, Kim I, Park M, Im SH, Shin K, Kim S (2022). Network-based machine learning approach to predict immunotherapy response in cancer patients. Nat Commun.

[35] Kazal LA, Spicer DS, Brahinsky RA (1948). Isolation of a crystalline trypsin inhibitor-anticoagulant protein from pancreas. J Am Chem Soc.

[36] Mehner C, Miller E, Hockla A, Coban M, Weroha SJ, Radisky DC, Radisky ES (2020). Targeting an autocrine IL-6-SPINK1 signaling axis to suppress metastatic spread in ovarian clear cell carcinoma. Oncogene.

[37] Ru N, Wu SY, Wang L, Zhu JH, Xu XN, Guo JY, Hu LH, Li ZS, Zou WB, Liao Z (2021). SPINK1 mutations and risk of pancreatic cancer in a Chinese cohort. Pancreatology.

[38] Li D, Zhang X, Ding Z, Ai R, Shi L, Wang Z, He Q, Dong Y, Zhu Y, Ouyang W (2022). Identification and exploration of serine peptidase inhibitor Kazal type I (SPINK1) as a potential biomarker correlated with the progression of non-small cell lung cancer. Cell Biochem Biophys.

[39] Räsänen K, Itkonen O, Koistinen H, Stenman UH (2016). Emerging roles of SPINK1 in cancer. Clin Chem.

[40] Flavin R, Pettersson A, Hendrickson WK, Fiorentino M, Finn S, Kunz L, Judson GL, Lis R, Bailey D, Fiore C (2014). SPINK1 protein expression and prostate cancer progression. Clin Cancer Res.

[41] Huang K, Xie W, Wang S, Li Q, Wei X, Chen B, Hua Y, Li S, Peng B, Shen S (2021). High SPINK1 expression predicts poor prognosis and promotes cell proliferation and metastasis of hepatocellular carcinoma. J Invest Surg.

[42] Gajewski TF, Schreiber H, Fu YX (2013). Innate and adaptive immune cells in the tumor microenvironment. Nat Immunol.

[43] Pitt JM, Marabelle A, Eggermont A, Soria JC, Kroemer G, Zitvogel L (2016). Targeting the tumor microenvironment: removing obstruction to anticancer immune responses and immunotherapy. Ann Oncol.

[44] Oura K, Morishita A, Tani J, Masaki T (2021). Tumor immune microenvironment and immunosuppressive therapy in hepatocellular carcinoma: a review. Int J Mol Sci.

[45] Llovet JM, Castet F, Heikenwalder M, Maini MK, Mazzaferro V, Pinato DJ, Pikarsky E, Zhu AX, Finn RS (2022). Immunotherapies for hepatocellular carcinoma. Nat Rev Clin Oncol.

[46] Leslie J, Mackey JBG, Jamieson T, Ramon-Gil E, Drake TM, Fercoq F, Clark W, Gilroy K, Hedley A, Nixon C (2022). CXCR2 inhibition enables NASH-HCC immunotherapy. Gut.

[47] Clevers H, Nusse R (2012). Wnt/β-catenin signaling and disease. Cell.

[48] He S, Tang S (2020). WNT/β-catenin signaling in the development of liver cancers. Biomed Pharmacother.

[49] Xu C, Xu Z, Zhang Y, Evert M, Calvisi DF, Chen X (2022). β-Catenin signaling in hepatocellular carcinoma. J Clin Investig.

[50] Kim W, Khan SK, Gvozdenovic-Jeremic J, Kim Y, Dahlman J, Kim H, Park O, Ishitani T, Jho EH, Gao B (2017). Hippo signaling interactions with Wnt/β-catenin and Notch signaling repress liver tumorigenesis. J Clin Investig.

[51] Wei S, Dai M, Zhang C, Teng K, Wang F, Li H, Sun W, Feng Z, Kang T, Guan X (2021). KIF2C: a novel link between Wnt/β-catenin and mTORC1 signaling in the pathogenesis of hepatocellular carcinoma. Protein Cell.

[52] Katoh M (2018). Multi-layered prevention and treatment of chronic inflammation, organ fibrosis and cancer associated with canonical WNT/β-catenin signaling activation (Review). Int J Mol Med.

[53] Li Q, Sun M, Wang M, Feng M, Yang F, Li L, Zhao J, Chang C, Dong H, Xie T (2021). Dysregulation of Wnt/β-catenin signaling by protein kinases in hepatocellular carcinoma and its therapeutic application. Cancer Sci.

